# How to Evaluate and Improve Foot Strength in Athletes: An Update

**DOI:** 10.3389/fspor.2019.00046

**Published:** 2019-10-11

**Authors:** Romain Tourillon, Boris Gojanovic, François Fourchet

**Affiliations:** ^1^Faculty of Sport Sciences, University of Nantes, Nantes, France; ^2^School of Physical Therapy and Rehabilitation, IFM3R, Saint-Sébastien sur Loire, France; ^3^Motion Analysis Lab, Physiotherapy and Sports Medicine Department, Swiss Olympic Medical Center, La Tour Hospital, Meyrin, Switzerland

**Keywords:** intrinsic foot muscles, foot strengthening, assessment, track and field athletics, exercises

## Abstract

The foot is a complex system with multiple degrees of freedom that play an essential role in running or sprinting. The intrinsic foot muscles (IFM) are the main local stabilizers of the foot and are part of the active and neural subsystems that constitute the foot core. These muscles lengthen eccentrically during the stance phase of running before shortening at the propulsion phase, as the arch recoils in parallel to the plantar fascia. They play a key role in supporting the medial longitudinal arch, providing flexibility, stability and shock absorption to the foot, whilst partially controlling pronation. Much of the foot rigidity in late stance has been attributed to the windlass mechanism – the dorsiflexion of the toes building tension up in the plantar aponeurosis and stiffening the foot. In addition, recent studies have shown that the IFM provide a necessary active contribution in late stance, in order to develop sufficient impedance in the metatarsal-phalangeal joints. This in turn facilitates the propulsive forces at push-off. These factors support the critical role of the foot in providing rigidity and an efficient lever at push-off. During running or sprinting, athletes need to generate and maintain the highest (linear) running velocity during a single effort in a sprinting lane. Acceleration and sprinting performance requires forces to be transmitted efficiently to the ground. It may be of particular interest to strengthen foot muscles to maintain and improve an optimal capacity to generate and absorb these forces. The current evidence supports multiple exercises to achieve higher strength in the foot, such as the “short foot exercise,” doming, toes curl, towing exercises or the more dynamic hopping exercises, or even barefoot running. Their real impact on foot muscle strength remains unclear and data related to its assessment remains scarce, despite a recognized need for this, especially before and after a strengthening intervention. It would be optimal to be able to assess it. In this article, we aim to provide the track and field community with an updated review on the current modalities available for foot strength assessment and training. We present recommendations for the incorporation of foot muscles training for performance and injury prevention in track and field.

## Introduction

The foot is a complex joint system with multiple degrees of freedom that play an important role in athletic tasks such as running or sprinting. The compliance of the foot is remarkable and its spring-like properties—in the medial longitudinal arch (MLA) – allow mechanical energy to be stored and returned at each step (Ker et al., 1987). Previous studies have shown that this spring mechanism was provided by the elastic components of the plantar fascia or aponeurosis (PA). This may account for 8–17% of the mechanical energy required for a stride (Ker et al., 1987; Stearne et al., 2016) and it increases stiffness via the windlass mechanism. Recent studies showed however that this spring cannot simply be passive, as it cannot explain the ability of the foot to adapt to the mechanical loads of running or sprinting (Riddick et al., 2019). Kelly et al. (2018) showed during running that as speed increases, so does the dissipation of mechanical energy within the foot. This can be modulated by the muscular capacity of the intrinsic foot muscles (IFM).

The IFM are the main local stabilizers of the foot and are part of the active and neural subsystems that constitute the foot core (McKeon et al., 2015). With their anatomical insertions located under the foot, these muscles lengthen eccentrically during the early stance phase of running, producing negative work before they shorten during the late stance phase as the arch recoils to produce positive work (Fourchet and Gojanovic, 2016). This active contraction aids propulsion and is enabled by the following three muscle-tendon units: abductor hallucis (AbH), flexor digitorum brevis (FDB), and quadratus plantae (QP) (Kelly et al., 2015).

Because the IFM are usually neglected in assessment and treatment, a key component of foot core stability is not considered. We aim to provide the track and field community an updated review on the current modalities available for foot strength assessment and training. Running or sprinting is a cyclic activity involving all joints and muscles groups in the lower limbs, including the IFM, and the metatarsophalangeal (MTP) joint. We believe that it is of interest to assess the MTP joint's role during sprinting, especially looking at the link between midfoot and plantar flexors' torque before considering the various strengthening modalities and protocols. We will present recommendations for the incorporation of foot muscles training or performance and injury prevention in track and field.

## The Forefoot Region and Running or Sprinting

During running or sprinting, athletes need to generate and maintain the highest (linear) running velocity during a single effort in a sprinting lane. Acceleration and sprinting performance requires forces to be transmitted efficiently to the ground. In particular, the production of horizontal force during the acceleration, more than vertical force, is related to sprinting performance. Indeed, the fastest runners at the end of the acceleration phase are not those who produce the highest total force, but those who manage to orient the forces horizontally (Morin and Samozino, 2016).

To achieve this, the sprinter must accomplish a series of segment rotations (Krell and Stefanyshyn, 2006) and gross moment generation about the lower limb joints, including hip, knee, and ankle (Tanaka et al., 2019). The small MTP joints (via dorsiflexion) may be related to sprint performance, as several studies have shown (Krell and Stefanyshyn, 2006; Bezodis et al., 2012). Stefanyshyn and Nigg (1997) investigated the energy patterns at the foot level during sprinting and found a large negative net energy balance (a lot of energy absorbed, whilst little produced) in the MTP joint during early to late stages of stance phase. These authors concluded that performance may be improved through a reduction in the energy loss at the MTP joint, but the question of how to achieve a better energy balance, hence better performance at push-off, remains. For example, Smith et al. (2014) found that varying the stiffness of spikes resulted in a significant decrease in MTP joint range of motion as well as dorsiflexion velocity when compared to barefoot. Spikes enable the MTP joint to plantar flex during push-off without affecting the windlass mechanism, which in turn facilitates propulsion by increasing the length of the moment arm (Smith et al., 2014).

This longer lever arm requires increased strength from the plantar flexors, and running athletes can benefit from it: a larger and more efficient horizontal force production may enhance performance (Morin and Samozino, 2016). A recent study showed that IFM activity in late stance is needed to generate sufficient impedance at the MTP joints, which in turn provides an efficient push-off (Farris et al., 2019).

Finally the MTP joint plays a key role during sprinting and that may result in the development of very strong foot muscles in sprinters population. However, Tanaka et al. (2019) demonstrated that although elite sprinters have thicker foot muscles compared with non-sprinters, AbH thickness correlates positively with their 100 m personal best. In other words a bigger AbH might be a negative factor for superior sprint performance. On the contrary, Yuasa et al. (2018) showed a significant correlation in collegiate American football players between maximal toe flexors strength with a dorsiflexed MTP and the ability to change direction in pro-agility and 3-cone tests. Abe et al. (2016) found in active subjects that fourth and fifth toe flexor strength was correlated positively with walking speed in men (*r* = 0.584) and women (*r* = 0.553), whereas Hashimoto and Sakuraba (2014) found that 8 weeks of toe flexors exercises decreased 50 m best personal time in 12 men.

Although toe flexors strength is generated by a simultaneous action of both intrinsic and extrinsic foot muscles, the IFM seem most likely to be the main contributors to MTP joints torque (Farris et al., 2019). Again, we should not underestimate the role of the MTP joints in strength training. In summary, strengthening interventions should not be limited to extrinsic foot and ankle muscles (e.g., flexor digitorum longus, triceps surae, flexor hallucis longus), but should also target intrinsic foot muscles.

## The Midfoot (medial) Region and Running or Sprinting

The foot enjoys some flexibility characterized by the medial longitudinal arch (MLA) which compresses and recoils. This ability allows mechanical energy to be stored and then released sequentially with every running step. Previous studies have proposed that mobility of the MLA can partly enhance the triceps surae and longus flexor hallucis moment during the push-off (Leardini et al., 2007; Kelly et al., 2014). Fourchet et al. (2015) emphasized the important role of the MLA in transmission of force through the foot and as a load-absorbing structure in fatigued adolescent runners. This highlighted the reciprocal interaction between plantar flexors and MLA – a compliant MLA results in hyperpronation and this may impede force transmission through the foot at stance phase leading to early plantar flexor fatigue. This means that a more flexible midfoot does not really lock, and hence produces less power, inefficient force transfer through the foot lever and insufficient foot stiffness.

This relationship between MLA and plantar flexor torque has been shown in one study where hyperpronated feet showed lower concentric force of the plantar flexors when compared to neutral feet (Snook, 2001). These findings gave support to the following biomechanical theory: the lever angle of the Achilles tendon and the plantar flexors would at a disadvantage in hyperpronation, which leads some force produced by these muscles to be applied medially (no propulsive effect) rather than mostly upward (Fourchet, 2012). From a neurophysiological perspective, studies on electromyography (EMG) activity and pronation showed that hyperpronation may alter the function of foot and ankle muscles. Novacheck (1998) reported a delayed time to maximum pronation beyond 40% of stance in case of excessive pronation. This could be explained by the decreased activity of plantar flexors during fatigue: their supination effect is decreased and causes an increased load under the MLA. Fourchet et al. (2015) shown after high intensity running that plantar flexors display a reduced resistance to fatigue and an increase in relative load medially under the midfoot. We hypothesized that excessive foot pronation leads to fatigue of plantar flexor and points to the interdependence between plantar flexors and IFM, the latter being often difficult to turn on (Boon and Harper, 2003). We propose that there may be an interdependent coupling between plantar flexors and IFM, which is made biomechanically possible as plantar flexion moves the center of pressure forward and increases the load under the midfoot.

Finally, a stiff MLA seems to play a key role in ensuring a stable stance phase, and facilitating its load-absorbing task, the one which mitigates the dissipation of the mechanical energy produced by plantar flexors at push-off. Takahashi et al. (2016) demonstrated in walking that an increased foot stiffness (with shoes and orthotics) altered soleus muscle behavior, resulting in greater peak force and reduced fascicle shortening speed. Therefore, in addition to the optimization of spikes conception, implementing strengthening exercises of the intrinsic foot musculature with the aim to improve strength and stiffness of the MLA appears to be of high interest in athletes. This brings us to the necessity of specific and validated tests to assess such interventions.

## Testing Foot Strength

The assessment of foot muscle strength is addressed in the literature with magnetic resonance imaging (MRI) or ultrasound imaging (USI) (Soysa et al., 2012; Gooding et al., 2016; Ridge et al., 2018) in order to quantify muscle thickness or cross sectional area, but these modalities are expensive and not applicable on the field by coaches or athletic trainers. Numerous other affordable measurement methods are available and come with an interesting level of validity and reliability. We can mention the toe flexor strength with toe-dynamometry (Spink et al., 2010) and the paper grip test (De Win et al., 2002) and there are several indirect tests assessing the strength or stiffness through the deformation or mobility of the foot arches in weight bearing and non-weight bearing conditions. They are the medial arch height (Okamura et al., 2017), the arch rigidity index (Mulligan and Cook, 2013), the navicular drop (ND), and the foot mobility measurement (FMM) (McPoil et al., 2009), which we will describe in detail.

### Medial Arch Height

This assessment performed during gait and/or standing phase requires the use of the Oxford Foot Model, a 3D multi-segment foot model with a good to excellent repeatability (Okamura et al., 2017). In this test, the MLA height is defined as the normal distance of the plane of the forefoot from the proximal first metatarsal marker by the Oxford Foot Model. The medial arch height can be measured before and after a strengthening programme or a fatiguing protocol for instance (Okamura et al., 2017).

### Arch Rigidity Index

**Figure 1:**
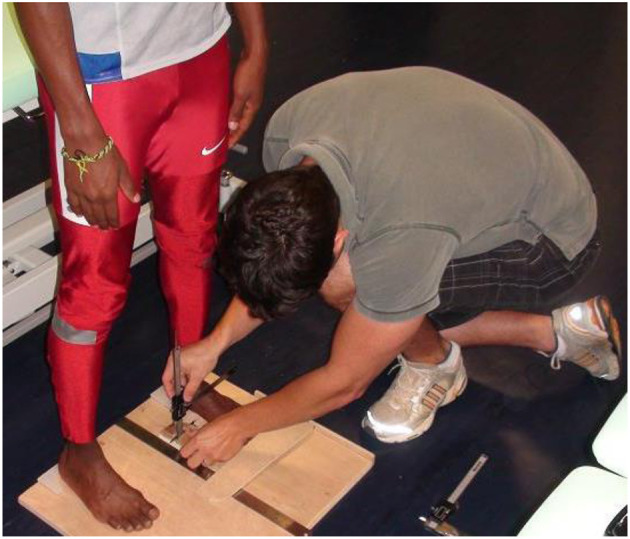
Measurement of the arch rigidity index.

The arch rigidity index (ARI) is calculated by dividing the standing arch height index by the sitting arch height index and it represents the structural mobility of the MLA (Mulligan and Cook, 2013). An ARI close to 1 represents a stiffer MLA while increasing foot flexibility correlates with numbers that rise well above 1. The arch height index is calculated by dividing the height of the dorsum of the foot by the truncated length of the foot to obtain a ratio in both seated and standing positions (Figure 1). The truncated length is the distance from the most posterior aspect of the calcaneus to the center of the first metatarsal head, and the height of the dorsum of the foot can be measured with a modified carpenter's square with a bubble level arm at 50% of the total foot length.

### Navicular Drop

The sit-to-stand double-leg or single-leg navicular drop test is the most popular evaluation of longitudinal arch stability in the literature. The athlete sits with his hips, knees, and ankles bent to 90° and the feet resting on the floor. The inferior border of the prominent tuberosity of the navicular bone is palpated and marked with a pen, and the distance to the ground is measured using a steel ruler (resolution: 0.5 mm). At this point the tester asks the athlete to stand barefoot on a 4-in (10.16 cm) box, full weight on the foot being measured, while the other foot rests lightly on the box (Cote et al., 2005). The difference between the two measures (sitting vs. standing) is the navicular drop. The tester must repeat three measures and the average value is recorded.

### Foot Mobility Measurement

The foot mobility measurement is a composite measure of vertical and medial to lateral mobility of the midfoot, whereas ND assess only vertical mobility. It has been described as a relevant technique for the assessment of foot mobility differences between non-weight bearing and weight bearing positions (McPoil et al., 2009).

To measure the FMM, three instruments are needed: weight bearing and non-weight bearing arch height gauges and a device to measure midfoot width, both of which can be relatively easily manufactured, with the inclusion of a digital caliper (Figure 1). Refer to McPoil et al. (2009) for a full description of the method. The athlete stands on a foot measurement platform (heels placed in heel cups) in order to measure dorsal arch height and midfoot width in bipodal weight bearing. Then, the same non-weight bearing measurements are recorded with the athlete sitting, both legs hanging in a perpendicular relaxed position. The procedure used for the weight bearing measures is then repeated. A method based on the Pythagorean Theorem is used to calculate the FMM:

(1) $$\begin{array}{r} \text{FMM}=\surd {\left(DiffAH\right)}^{2}+{\left(\text{DiffMFW}\right)}^{2} \end{array}$$

where “Diff AH” and “Diff MFW” are the changes in dorsal arch height and in midfoot width between weight bearing and non-weight bearing, respectively.

## Foot Strengthening Strategies

Strengthening of the foot muscles responds to the same training principles as any other muscle group. IFM strengthening can be performed in isometric, concentric, eccentric or plyometric modes.

### Isometric Strengthening: Short Foot Exercise, Toe-Posture Exercises, and Tower Curl

**Figure 2:**
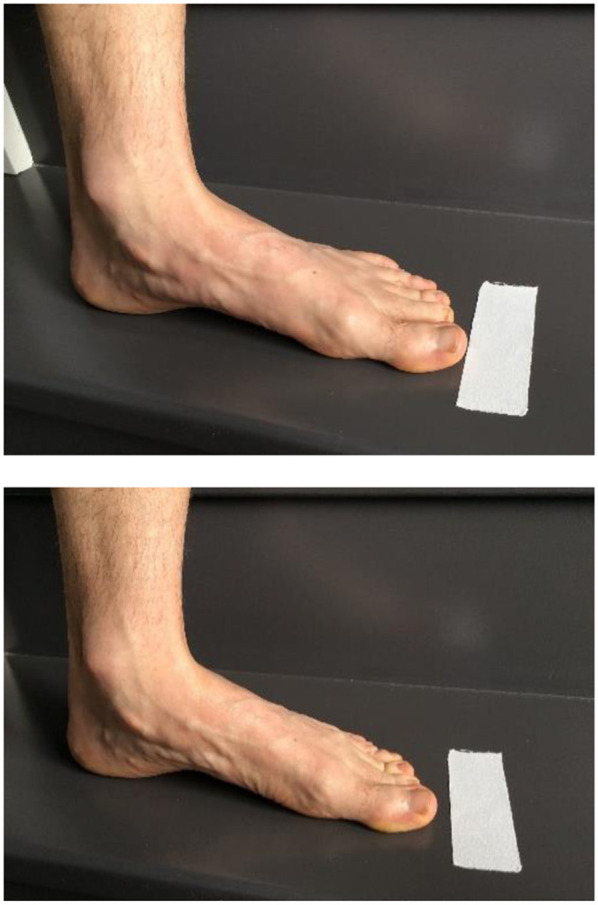
Short foot exercise.

In isometric strengthening of the IFM, the most recognized exercise is the short foot exercise (SFE) (McKeon and Fourchet, 2015), where volitional control of the intrinsic foot muscles elevates the foot arches and shortens the foot. This is described as part of the foot core paradigm introduced by McKeon et al. (2015). The SFE is typically challenging to teach and learn, so that three gradual training steps have been recommended: (1) passive mode, (2) active-assisted mode, and (3) active mode. In passive mode, the athlete's foot is moved by the therapist or the coach through the short foot movement, allowing the athlete to feel, learn, and integrate the different positions. In the active-assisted mode, active contractions of the plantar IFM are added to actively obtain the short foot position. Finally the active mode consists in the athlete performing the exercise without assistance (Figure 2).

**Figure 3:**
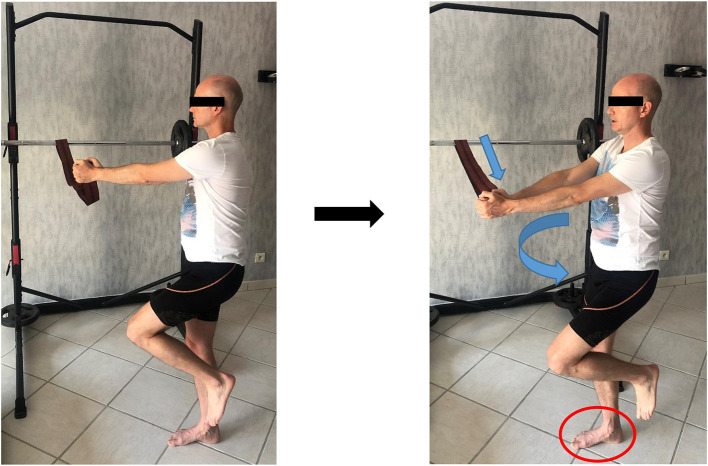
Short foot exercise with cross-body inversion focus (with written informed consent obtained from the subject).

It is also possible to combine the SFE with active and resisted activities of the upper body in order to create a cross-body inversion focus (i.e., trunk and pelvis medial rotation) and promote muscular chains facilitations (Figure 3).

**Figure 4:**
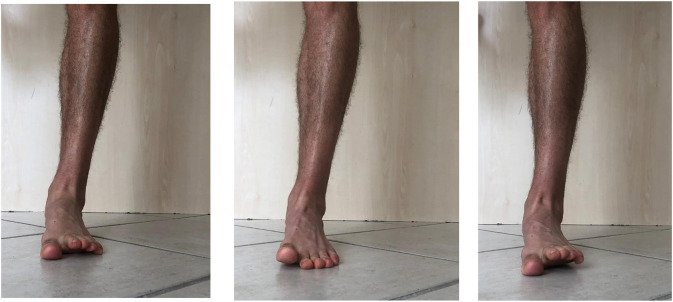
Toe spread out exercise, 1st toe extension and 2nd to 5th toe extension.

It is worth mentioning that numerous others exercises commonly referred to as “toe yoga” or “toe posture exercises” have been shown to activate the IFM in a isometric contraction (Table 1). For example, the Toe-Spread-Out exercise (TSO), First To Fifth toe extension exercises are validated by Gooding et al. (2016). The TSO is carried out by a sequential extension of all toes, followed by hallux abduction, hallux flexion, and fifth toe flexion (Figure 4).

**Table 1:** Summarizes the recent studies on the topic.

**Authors**	**Population**	**Group exercises**	**Modalities**	**Results**
Sulowska et al. (2019)	Long distance runners (*n* = 47)	Vele forward lean + Reverse tandem gait + Short foot exercise + ankle muscles strengthening for: neutral (group 1) and pronator (group 2)	6 weeks 30 min daily Progression (every 2 weeks): increasing load and level of difficulty and adding perturbation (tennis ball, a stability disc, and band loops)	↑ peak torque knee flexion (group 2) ↑ power in each 35 m run (group 2) ↓ 35 m run time (group 2)
Unver et al. (2019)	Pes planus (*n* = 41)	Short Foot Exercise (SFE) group Control group	6 weeks 2 times/week (supervision)/5 times/week (home) 5 s of contraction 3 sets of 15 repetitions Progression: sitting position (1 et 2), double (3 et 4) and single leg stance (5 et 6)	↓ Navicular Drop (ND), Foot Posture Index (FPI), Pain and Disability Score ↑ Plantar force at midfoot region
Fraser and Hertel (2018)	Healthy, recreationally active young adults (*n* = 24)	IFM exercises program: Hallux extension Lesser toe extension Toes-Spread-Out (TSO) SFE	4 weeks 3 times daily Completed a daily training log detailing the type, position, volume, and frequency of exercises performed. Home exercises were progressed if the task was performed adequately (no compensations, not too slow, and no obvious clumsiness).	↑ IFM activation ↓ perceived difficulty No significant (NS) effect on muscle activation
Taddei et al. (2018)	Healthy long distance runners (*n* = 31)	Foot and Ankle muscle strength training group Stretching group	8 weeks 2 times/week and then 3 times/week (8 weeks) and 1 year of follow-up 20–30 min (guided by software videos)	↑ cross sectional area (CSA) of AbH and FDB No effect on IFM strength Improvement for some foot kinematics parameters
Sudhakar et al. (2018)	Middle distance runners (*n* = 30)	Vele forward lean + Walking backward (Reverse tandem gait) for VFR group Plantar Short Foot (PSF) Exercises group: TSO + Plantar roll out exercises + Fine toe curl exercises and big toe curl exercises	4 weeks 5 times/week 15 min, 2 times/day Progression = > Sitting position, standing position, half squat	↑ of Functional Movement Screen (FMS) compared to VRF group ↓ Foot posture index
Gooding et al. (2016)	Healthy subjects (*n* = 8)	Hallux extension Lesser toe extension Toe Spread Out (TSO) SFE	1 set of 40 repetitions	SFE ↑ activation of AbH (29.7%) and FDB (29.8%)
Kamonseki et al. (2016)	Plantar fasciitis (*n* = 83)	Foot exercise group Foot and Hip group Stretching alone exercise group	Foot exercise group: Toe curl exercise (3 sets of 15 reps): 1–2 kg SFE (3 times for 1 min)	All 3 exercise groups improve: Quality of life, pain, activities of daily living, sports & recreation
Kim and Kim (2016)	Flexible flat foot (*n* = 14)	SFE group Arch support insoles group	30 min per day 3 times/week during 5 weeks	↑ Y Balance test (both group) ↓ Navicular drop (SFE group)
Sulowska et al. (2016)	Long distance runners (*n* = 25)	Vele forward lean + Reverse tandem gait + SFE	6 weeks Daily basis for 30 min Progression: sitting, standing, half-squat	↓ FPI: item 1 et item 3 ↑ FMS (deep squat, active straight leg raise)
Kim et al. (2015)	Mild and moderate hallux valgus (*n* = 12)	Toe spread out + Orthosis	20 min/days during 8 weeks 4 times per week	↓ hallux valgus angle (HVA) + HVA during active abduction ↑ CSA AbH
Panichawit et al. (2015)	Flexible flat foot (*n* = 5)	Calf muscles stretching exercise, strengthening of the tibialis posterior (TP), Peroneus Longus (PL), Flexor Digitorum Longus (FDL), ankle dorsiflexion, and IFM as well as co-contraction of the invertors and evertors muscles	Stretching: 10 reps Strengthening: 10–15 reps (3 sets) Progression: resistive exercises with added bands	↑ TP and PL strength ↓ Foot function score NS difference in plantar contact area and plantar peak pressure
Hashimoto and Sakuraba (2014)	Healthy male subjects (*n* = 12)	Toe flexor strength	8 weeks 200 reps/day, 3 times per week Load (3 kg -> 10 kg)	↑ vertical jump height + 50 m dash performance + IFM strength + 1 legged long jump ↓ arch length
Moon et al. (2014)	Hyperpronated feet (*n* = 18)	SFE	1 session: 5 sets of 3 reps × 5 (2 min rest) with 5 s of contraction	↑ dynamic balance
Goldmann et al. (2013)	Healthy subjects (*n* = 15)	Toe flexor strength	7 weeks (560 contractions) 90% of maximal voluntary isometric contraction	↑ toe strength ↑ horizontal jump distance ↑ external MTP joint dorsiflexion moments ↑ MTP plantar flexion moment
Kim et al. (2013)	Mild hallux valgus (*n* = 25)	TSO group SFE group	Practice for 2 weeks SFE and TSO exercises were conducted for 15 min once per day On the day of the experiment, subjects performed the SFE and TSO exercises 5 times for familiarization with both exercises.	TSO exercise showed significantly greater activation of the AbdH than did SFE ↑ ratio of AbdH to AddH muscle activity significantly higher in TSO group Significantly Greater angle of the first MTP joint in horizontal plane during TSO than SFE
Mulligan and Cook (2013)	Healthy subjects (*n* = 21)	SFE	4 weeks 3 min/day 30 reps (5 s of contraction) Progression: sitting to double and single leg stance + perturbations (through instability or vision)	↓ navicular drop ↑ Arch height index Improvement in balance and reach task
Lynn et al. (2012)	Healthy subjects (*n* = 24)	SFE group Tower curl group	4 weeks 100 reps/day 5 s of contraction Progression: sitting (week 1 and 2), standing (week 3 and 4)	NS difference in navicular height or static balance test ↓ Medio-lateral center of pressure movement in dynamic balance test (SFE > for non-dominant limb)
Jung et al. (2011b)	Pes planus (*n* = 28)	Foot orthosis + SFE group Orthosis group	8 weeks 3 sets of 15 reps (2 times/week): hold the position for 5 s with 2 min rest periods between sets Progression: increased up to 5 reps and then in the next progression, the holding time increased to 10 seconds	↑ CSA AbH in foot orthosis + SFE group ↑ flexor hallucis strength in SFE + Orthosis group
Jung et al. (2011a)	Normal feet (*n* = 20)	SFE group Tower curl group	SFE or Tower curl in maximal contraction (3 trials of 5 s = > muscular activation) 15 min/day during 2 weeks Progression: sitting and standing on single leg	↑ AbH activity in SFE group in comparison to Tower curl group

The “First-Toe Extension” or “Hallux-Extension” exercise is performed by extending the first metatarsophalangeal joint while maintaining the lesser toes (Second To Fifth) in contact with the floor (Figure 4). The “Second- To Fifth-Toes Extension” or “Lesser-Toes-Extension” exercise consists in extension of toes 2–5 whilst maintaining the hallux in contact with the ground (Figure 4).

**Figure 5:**
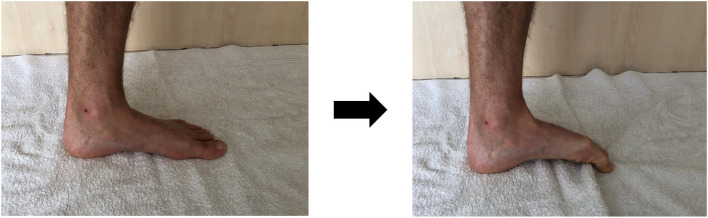
Tower curl (Toe flexor exercise).

We have stressed the importance of the MTP joint for sprint performance and some MTP strength exercises can be discussed. Previously used techniques that attempted to strengthen the IFM involved toe-flexion exercises (Hashimoto and Sakuraba, 2014) but it seems that these exercises recruit more of the extrinsic foot musculature (such as the flexor digitorum longus) and make these muscles dominant over the IFM (Lynn et al., 2012). Hashimoto and Sakuraba (2014) developed a strength training program that focused on IFM strength by excluding the extrinsic muscles as much as possible by bringing the ankle in plantar flexion. The “Towel curl exercise” or “toe flexor strength” is performed in sitting or standing position with or without added weights, where the subject is asked to slowly curl the toes and fold the towel or dynamometer under the foot by flexing the toes [interphalangeal (IP) and metatarsophalangeal (MTP) flexion] (Figure 5). This type of exercises permits also to work the horizontal strength product by the IFM with the help of the extrinsic foot muscles.

### Dynamic and Plyometric Foot Strengthening

**Figure 6:**
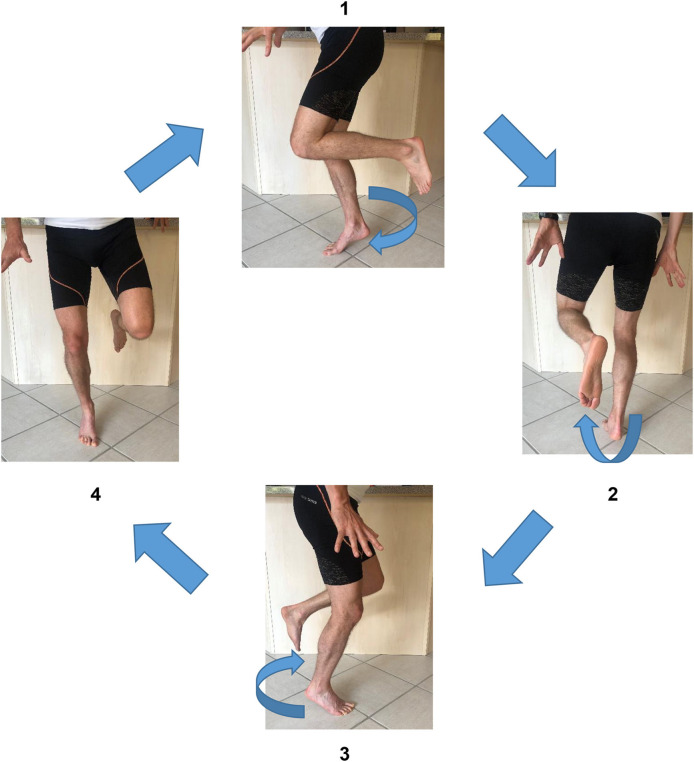
Short foot exercise in rotation (with written informed consent obtained from the subject).

**Figure 7:**
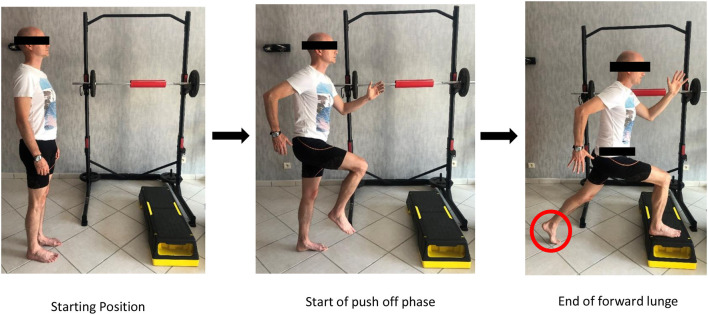
Short foot exercise during propulsion (with written informed consent obtained from the subject).

From a biomechanical perspective, isometric exercises are not reflective of how foot muscles work during locomotion (Farris et al., 2019). The magnitude of load at the midfoot during running or even walking is so high that it is not possible for the foot muscles to generate sufficient force with low load tasks like the SFE. We suggest to progress from isometric to plyometric exercises in order to get closer to the specific function of running. We can consider heel rises or any exercises shifting the CoP in front of the body, as these will likely impose a much higher load on the midfoot (Figures 6, 7).

### Minimalist or Barefoot Running

The literature on the effects of running on foot muscular adaptations is relatively scarce and somewhat contradictory. Nevertheless, it does suggest that running could improve the cross-sectional area and the volume of foot muscles, and that this may be modulated by running mileage and experience (Miller et al., 2014; Johnson et al., 2015; Chen et al., 2016; Ridge et al., 2018). Based on the limited evidence available, there seems to be a positive effect of running on intrinsic muscle strength and size (Garofolini and Taylor, 2019). We will review some of these studies briefly.

Johnson et al. (2015) found that 10 weeks of training in minimal running shoes may be effective in increasing muscle size, especially abductor hallucis cross-sectional area. Chen et al. (2016) found that a 6-months transitioning running program to minimal shoes led to larger IFM. It is worth mentioning that a strengthening program was added in this protocol, and it is not possible to say which component was responsible for the observed changes in muscle volume (Chen et al., 2016). Similarly, after a 12 weeks transitioning programme, a significant improvement was reported in the volume and the cross-sectional area of the abductor digiti minimi in recreational runners (Miller et al., 2014).

### Neuromuscular Electrical Stimulation (NMES)

An additional modality for the volitional strengthening of foot muscles is neuromuscular electrical stimulation (NMES) of the IFM. The aim is to strengthen the foot lever as the first interface between the ground and the athlete (Fourchet and Gojanovic, 2016). The incorporation of this modality has been shown to improve foot postural control and plantar pressure distribution in runners (Fourchet et al., 2009, 2011).

Scientific findings suggest that using NMES on foot muscles can decrease navicular drop after a 3-weeks programme (three sessions a week). In another study, we showed that combining NMES with other exercises during 5 weeks shifted plantar foot pressure distribution laterally, which resulted in a reduction of loads under the medial midfoot during running (Fourchet et al., 2009, 2011).

**Figure 8:**
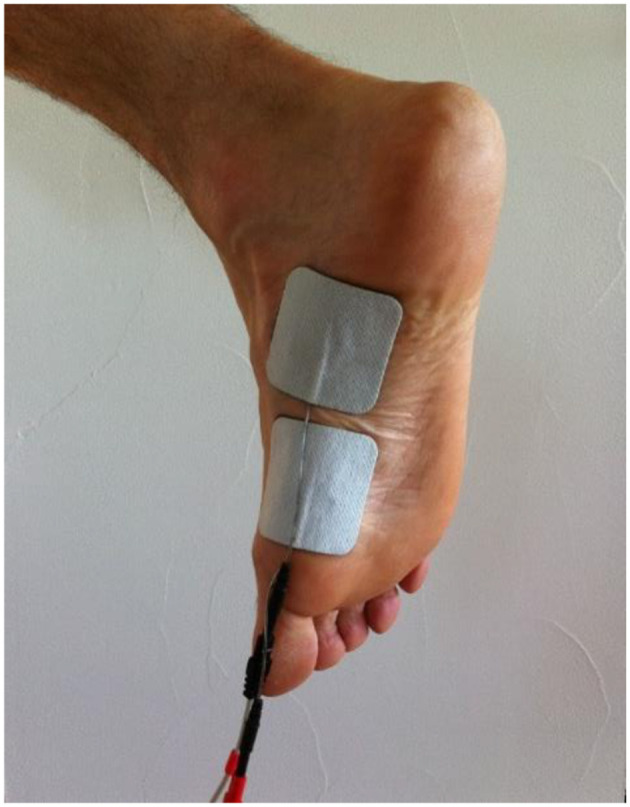
Placement of electrodes for NMES on medial arch intrinsic foot muscles (McKeon and Fourchet, 2015).

Practically speaking, the set-up is very simple: the athlete stands with feet on the ground whilst the stimulator delivers NEMS as for 15 min, and a total of ~75 contractions completed per training session. The two electrodes are placed behind the head of the first metatarsal to stimulate the medial arch intrinsic muscles (Figure 8). Biphasic symmetric regular-wave pulsed currents (85 Hz) lasting 400 ms are delivered, and each tetanic stimulation is delivered for 4 s, followed by an active rest period lasting 8 s (McKeon and Fourchet, 2015).

We recommend that the athletes performs an average of 9 to 12 NMES sessions through 3–5 weeks, as this will bring effective improvements. The athlete begins in bipodal stance during the first sessions and then progresses to single-leg stance and plyometric activities such as hopping.

## Conclusion

The biomechanical specificities at the forefoot and the midfoot regions during running or sprinting require a high level of strength from the small foot muscles. The foot core system must act as a strong and rigid lever in order to best transfer lower limbs forces during propulsion, and it must also cope with significant amounts of constraints at the absorption phase, in the sense of impact attenuation.

The existing medical and scientific literature can help coaches and athletes to set up the most adapted exercises in order to strengthen their feet: variation and progression is necessary and ranges from isometric, concentric to eccentric contraction modes, from analytic to functional exercises, from volitional to electrically-assisted (NMES).

In order to track foot strength development and response to training, the athletic community can rely on several reliable and field-friendly assessments modalities.

This paper discusses aspects that are more performance-oriented or training-oriented, but the readers should keep in mind that the optimal control of the foot at stance phase is essential for the athlete's health as well. Overuse injuries linked to the control of the arch of the foot may be related to deficits in active foot stabilization during running, which may lead to increased tissue stresses. Medial tibial stress syndrome or Achilles tendinopathy are often linked to a lack of stiffness in the medial arch of the foot and its ability to cope with the changing demands for dynamic foot control.

We fully acknowledge that expert track and field coaches already apply a large body of the knowledge described in this article in their daily work with athletes. We do believe that for optimal health and performance outcomes, a close collaboration between coaches, sport scientists and medical staff is of primary interest and enables all parties to keep learning from each other.

## Author Contributions

All authors listed have made a substantial, direct and intellectual contribution to the work, and approved it for publication.

### Conflict of Interest

The authors declare that the research was conducted in the absence of any commercial or financial relationships that could be construed as a potential conflict of interest.
